# Can Ashwagandha Benefit the Endocrine System?—A Review

**DOI:** 10.3390/ijms242216513

**Published:** 2023-11-20

**Authors:** Michał Wiciński, Anna Fajkiel-Madajczyk, Zuzanna Kurant, Dominik Kurant, Karol Gryczka, Michal Falkowski, Magdalena Wiśniewska, Maciej Słupski, Jakub Ohla, Jan Zabrzyński

**Affiliations:** 1 Department of Pharmacology and Therapeutics, Faculty of Medicine, Collegium Medicum in Bydgoszcz, Nicolaus Copernicus University, M. Curie Skłodowskiej 9, 85-094 Bydgoszcz, Poland; aniafajkiel1@gmail.com (A.F.-M.); zuzannakurant@gmail.com (Z.K.); dominokurant1@gmail.com (D.K.); 98.karol@gmail.com (K.G.); 2 Department of Medicinal Chemistry, Faculty of Pharmacy, Collegium Medicum in Bydgoszcz, Nicolaus Copernicus University in Toruń, Dr. A. Jurasza 2, 85-089 Bydgoszcz, Poland; m.falkowski@cm.umk.pl; 3 Department of Oncology and Brachytherapy, Faculty of Medicine, Collegium Medicum in Bydgoszcz, Nicolaus Copernicus University in Toruń, Dr I. Romanowskiej 2, 85-796 Bydgoszcz, Poland; magda_piatkowska@o2.pl; 4 Department of Clinical Oncology, Professor Franciszek Lukaszczyk Oncology Center, Dr I. Romanowskiej 2, 85-796 Bydgoszcz, Poland; 5 Department of Hepatobiliary and General Surgery, Faculty of Medicine, Collegium Medicum in Bydgoszcz, Nicolaus Copernicus University, M. Curie Skłodowskiej 9, 85-094 Bydgoszcz, Poland; maciej.slupski@cm.umk.pl; 6 Department of Orthopedics and Traumatology, Faculty of Medicine, Collegium Medicum in Bydgoszcz, Nicolaus Copernicus University, M. Curie Skłodowskiej 9, 85-094 Bydgoszcz, Poland; jakub.ohla@cm.umk.pl (J.O.); jan.zabrzynski@cm.umk.pl (J.Z.)

**Keywords:** Ashwagandha, endocrine system, HPA axis, HPG axis, thyroid, reproductive system

## Abstract

*Withania somnifera*, also known as Ashwagandha, has been used in traditional medicine for thousands of years. Due to the wide range of its activities, there has been interest in its possible beneficial effects on the human body. It is proved that, among others, Ashwagandha has anti-stress, anti-inflammatory, antimicrobial, anti-cancer, anti-diabetic, anti-obesity, cardioprotective, and hypolipidemic properties. Particularly interesting are its properties reported in the field of psychiatry and neurology: in Alzheimer’s disease, Parkinson’s disease, multiple sclerosis, depression, bipolar disorder, insomnia, anxiety disorders and many others. The aim of this review is to find and summarize the effect that Ashwagandha root extract has on the endocrine system and hormones. The multitude of active substances and the wide hormonal problems faced by modern society sparked our interest in the topic of Ashwagandha’s impact on this system. In this work, we also attempted to draw conclusions as to whether *W. somnifera* can help normalize the functions of the human endocrine system in the future. The search mainly included research published in the years 2010–2023. The results of the research show that Ashwagandha can have a positive effect on the functioning of the endocrine system, including improving the secretory function of the thyroid gland, normalizing adrenal activity, and multidirectional improvement on functioning of the reproductive system. The main mechanism of action in the latter appears to be based on the hypothalamus–pituitary–adrenal (HPA) axis, as a decrease in cortisol levels and an increase in hormones such as luteinizing hormone (LH) and follicle-stimulating hormone (FSH) in men were found, which results in stress level reduction and improvement in fertility. In turn, other studies prove that active substances from *W. somnifera*, acting on the body, cause an increase in the secretion of triiodothyronine (T3) and thyroxine (T4) by the thyroid gland and a subsequent decrease in the level of thyroid-stimulating hormone (TSH) in accordance with the hypothalamus–pituitary–thyroid (HPT) axis. In light of these findings, it is clear that Ashwagandha holds significant promise as a natural remedy for various health concerns, especially those related to the endocrine system. Future research may provide new insights into its mechanisms of action and expand its applications in both traditional and modern medicine. The safety and toxicity of Ashwagandha also remain important issues, which may affect its potential use in specific patient groups.

## 1. Introduction

Adaptogens are nontoxic substances, often of plant origin, that restore normal physiological functioning when under stress or other unfavorable factors. One of them is Ashwagandha. As a plant well known around the world, it functions under different names. It is well known as *Withania somnifera*, which means “sleep inducer” in Latin, which correlates with its sleeping properties; Indian ginseng, widely used in Indian medicine; or Indian winter cherry [1]. With regard to the name of Ashwagandha itself, in Sanscrit (the sacred language of Hinduism), “ashwa” means horse and “gandha” means wet, what is supposed to be related with the smell of the roots. Other sources indicate that the name comes from its properties—ingested Ashwagandha grants horse-like strength [2]. Due to the place of its native occurrence (India, Sri Lanka, Baluchistan, Pakistan, Medditeranean regions, the Cannaries and Cape of Good Hope) and multidirectional activity, the plant has been widely used in traditional medicine of these regions for centuries [3,4]. Currently, it is proved that Ashwagandha has anti-stress, anti-inflammatory, antimicrobial, anti-cancer, anti-diabetic, anti-obesity, cardioprotective, and hypolipidemic properties [5]. Particularly interesting are its properties reported in the field of psychiatry and neurology: in Alzheimer’s disease, Parkinson’s disease, multiple sclerosis, depression, bipolar disorder, insomnia, anxiety disorders and many others [1,6,7,8,9]. *W. somnifera* active substances also became popular during the SARS-CoV-2 pandemic, due to its potential to inhibit coronavirus [10,11].

Also, Ashwagandha’s influence on the hormonal system deserves attention. It has been proved that it modulates pituitary functions [12], can improve thyroid gland homeostasis [13], regulates adrenal activity and has a multidirectional influence on the reproductive system [12,14].

The question remains, what makes Ashwagandha so multifunctional? This is due to the compounds it contains. Its roots, leaves, fruits and other plant parts are rich in active substances. Many studies described over 50 identified substances of Ashwagandha, among them alkaloids, flavonoids, steroidal lactones, N-containing compounds, steroids, and salts [1,15,16].

The aim of this review is to find and summarize the effect that Ashwagandha root extract has on the endocrine system and hormones. The multitude of active substances and the wide hormonal problems faced by modern society sparked our interest in the topic of Ashwagandha’s impact on this system. In this work, we also attempted to draw conclusions as to whether *W. somnifera* can help normalize the functions of the human endocrine system in the future.

## 2. Ashwagandha’s Active Substances and Their Biological Effects

*W. somnifera*, popularly known as Ashwagandha, is famous for its anti-inflammatory [17], anti-cancer [18,19,20], anti-depressant, anti-anxiety and insomnia-treating properties [21]. In addition, there are scientific reports supporting the use of Ashwagandha in the treatment of infertility and hormonal disorders [12,16]. Ashwagandha’s remarkable medicinal properties are undoubtedly due to its wealth of active substances, which demonstrate a variety of actions. The different biological species of the *Withania* genus also differ in the content of these substances. This plant is abundant in ingredients such as alkaloids, phenols, flavonoids, saponins, tannins, carbohydrates, steroidal lactones, β-sitosterol, scopoletin, sitoindosides, somniferiene, somniferinine, pseudotropine, anaferine, anahygrine, cysteine, chlorogenic acid, cuscohygrine, withanine, withaferine, withanolides, withananine, tropanol, 6,7β-epoxywithanon, and 14-α-hydroxywithanone [22]. Different parts of the plant vary in the composition of chemical compounds [2]. For example, the leaves contain five unidentified alkaloids, 12 withanolides, many free amino acids, chlorogenic acid, glycosides, glucose, condensed tannins and flavonoids. In addition, the roots are rich in alkaloids, amino acids, steroids, volatile oil, starch, reducing sugars, glycosides, hentriacontane, dulcitol and withaniol [23]. It seems that this wealth of active substances is not yet fully understood and still requires further research. Table 1 shows the classification of the active substances found in Ashwagandha.

**Figure 1:**
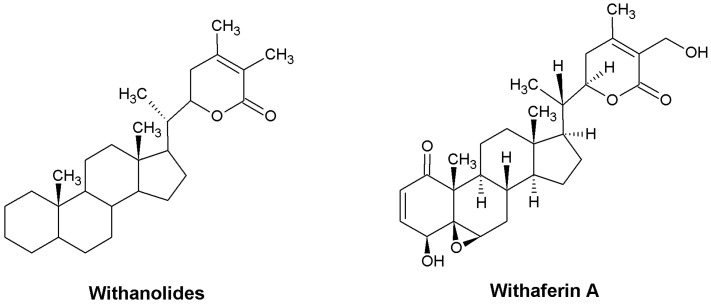
Structures of withanolides and withaferin A.

Withanolides and phenolic compounds are mainly responsible for the special medicinal properties of *W. somnifera* and show immune system-activating and antioxidant effects, respectively [23]. Structurally, withanolides belong to the 28-carbon atoms of natural steroidal lactones, built on an ergostane skeleton in which C-22 and C-26 are oxidized to form six- or five-membered lactone rings. These compounds are also known as 22-hydroxy ergostane-26- oleic acid-26, 22-lactone. A characteristic feature of withanolides is that the C8 or C9 side chains exhibit a lactone or lactol ring. The lactone ring can be five- or six-membered and can be linked to the carbocyclic group of the molecule via a C-C bond or via an O-bridge [22]. Of the withanolides isolated to date, withaferin A has received the most interest from researchers. It is found abundantly in *W. somnifera* and has great therapeutic potential—studies have shown that it has strong anti-inflammatory and anti-cancer activities [24]. Structurally, withaferin A, or (4β,5β,6β,22*R*)-4,27-dihydroxy-5,6:22,26-diepoxyergosta-2,24-diene-1,26-dione, is a 28-carbon withanolide with an ergostane and a δ-lactone structure [24]. It has two hydrogen bond donors and six hydrogen bond acceptor groups, which make it highly reactive [24]. Analysis of the chemical structure of withaferin A showed three positions that can interact with target proteins. Alkylation and nucleophilic binding reactions occur through the A ring at the C3 position and the epoxide at C24 [25]. Figure 1 shows the general chemical structure of withanolides and withaferin A.

In addition to withanolides, to which most research is currently dedicated, it is worth mentioning the remarkable properties of the phenolic compounds and alkaloids present in *W. somnifera*. Studies have shown that phenolic compounds in Ashwagandha have strong antioxidant and antibacterial properties, but the presence of these compounds determines the chemical composition of the formulation due to the origin of the raw materials and the plant part used, as well as the extraction protocol [23]. It was proved that water extracts, which showed higher antioxidant and antibacterial activity than water–methanol extracts, were particularly rich in polyphenols and ascorbic acid. On the other hand, water–methanol extracts showed higher inhibitory activity against acetylcholinesterase (AChe) than aqueous extracts, which inhibited butyrylcholinesterase (BChe) more strongly. These results suggest that there is a need to develop appropriate extraction methods to maximize the potential therapeutic properties of *W. somnifera* [23]. The alkaloids contained in *W. somnifera* extracts have anti-inflammatory properties, and this effect is greater than that caused by withanolides. In addition, alkaloids are characterized by lower toxicity [26]. Furthermore, anaferine and anahygrine, two alkaloids found in Ashwagandha, have been studied for anti-tuberculous properties and the development of new drugs against *Mycobacterium tuberculosis*. The obtained molecular docking results confirm their promising properties against the causative agent of human tuberculosis [27]. In silico studies have also shown that these substances may have neuroprotective properties and could potentially be used in the treatment of Alzheimer’s, Parkinson’s and other neurodegenerative diseases [28,29,30].

When presenting the healing properties of Ashwagandha, it is important to mention the wide range of molecular mechanisms that it activates. One of the best-known compounds present in *W. somnifera*—withanolides, mainly withaferin A, withanolide D and withanone—exhibit antioxidant properties by increasing the activity of cellular antioxidant enzymes (superoxide dismutase—SOD; catalase—CAT; glutathione S-transferase—GST; haem oxygenase 1—HO-1; NAD(P)H—quinone dehydrogenase 1) and a transcription factor—Nuclear factor E2-related factor 2 (Nrf2). Moreover, they have anti-inflammatory effects by inhibiting prostaglandin (PGE2) synthesis by cyclooxygenase-2 (COX-2), nitric oxide production by inhibition of nitric oxide synthase (iNOS), the release of interleukins (IL-6 and IL-1β) and nuclear factor κ light chain of activated B cells (NFκB) [12]. Withanolides also exhibit apoptosis-inducing activity through the stimulation of a series of factors, such as the production of reactive oxygen species, BCL2-related protein X (Bax), death receptor 5 (DR5), mitogen-activated protein kinase (MAPK), protease-activated receptor 4 (PAR-4), tumor suppressor proteins (p53 and p21) and major caspases (-3, -8, -9). By regulating cell division via cyclin-dependent kinases (CDKs), signal transducer and activator of transcription 3 (STAT3), B-cell lymphoma 2 (Bcl2), heat shock protein 90 (Hsp90), epidermal growth factor receptor (EGFR), human epidermal growth factor receptor 2 (HER2), inhibitor of the β subunit of nuclear factor κB kinase (IκKβ), proliferating cell nuclear antigen (PCNA) and survivin, these compounds also prevent cell proliferation. Furthermore, studies have shown that *W. somnifera* root extract induces effects on the central nervous system, including modulation of acetylcholinesterase activity, serotonin receptors and GABA (gamma aminobutyric acid) activity [12]. *W. somnifera* mainly affects the hypothalamic–pituitary–gonadal (HPG) axis through non-oxidative mechanisms, as well as anti-stress effects through the hypothalamic–pituitary–adrenal (HPA) axis [12]. The docking results support the hypothesis that withaferin A has the potential to inhibit the formation of the active NEMO/IKK complex. These studies suggest that withaferin A is a potent anti-tumor agent, as confirmed by its strong ability to modulate NF-κB [31]. Based on the results of the docking studies, it was noted that withaferin A showed beneficial binding to VEGF, and the results were highly comparable to the commercially available drug bevacizumab, demonstrating the ability to control angiogenesis, solid cancer growth and metastasis [32]. Structural modifications of withaferin A affect multiple signaling pathways, which contributes to improved therapeutic outcomes in various diseases. Studies have shown pharmacological activity of withaferin A against many neurodegenerative diseases [28,33], resulting from the inhibition of the enzymes acetylcholesterinase and butyrylcholinesterase, anti-diabetic effect by increasing the level of adiponectin and preventing the phosphorylation of peroxisome proliferator-activated receptors (PPARγ), cardioprotective effect by activating AMP-activated protein kinase (AMPK) and suppressing mitochondrial apoptosis [25].

Interestingly, molecular docking analysis has showed that, for example, withaferin A and withanone had strong inhibition potential of Penicillin-Binding Protein 4 (PBP4) and Ashwagandha extract had antibacterial properties [34]. Moreover, recent studies have shown that withaferin A and withanone present in Ashwagandha extract can covalently inhibit the SARS-CoV-2 M^pro^ protease [35], which is essential for replication and is the main target of antiviral drugs. It is also worth mentioning the possibility of withaferin A and withanone to interact with transmembrane serine protease 2 (TMPRSS2) and block the entry of SARS-CoV-2 into cells [36].

Ashwagandha is used not only in dietary supplements, but also in cosmetic products for skin care. It is believed that Ashwagandha root can be used to treat leukoderma, ulcers, scabies, as well as to heal skin wounds and reduce swelling. It is suggested that withaferin A, due to its anti-inflammatory properties, can be used in dermatological diseases, such as scleroderma or pigmentation disorders [37].

The potential therapeutic effects of the active substances discussed above are shown in Table 2.

Despite its numerous health-promoting properties, the active substances present in Ashwagandha extract may also cause toxic effects. There have been reports of Ashwagandha-induced liver injury that is typically cholestatic or mixed with severe jaundice and pruritus [38]. Siddiqui et al. found potential mechanism of liver damage by Ashwagandha in which withanone can form labile adducts with nucleosides deoxyguanosine (dG), deoxyadenosine (dA) and deoxycytidine (dC), thus interfering with the biological properties of DNA. Moreover, it also forms adducts with amines, which is a reversible process. Withanone is detoxified by glutathione (GSH), at limited levels of which it can induce DNA damage [39].

**Figure 2:**
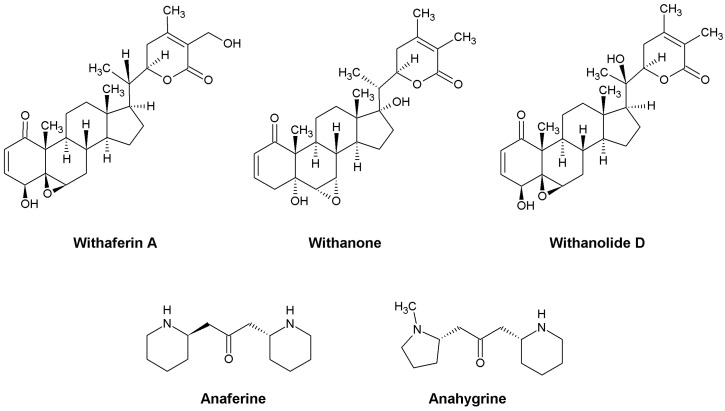
Structures of some active substances found in Ashwagandha.

Not all molecular mechanisms of Ashwagandha compounds have been discovered yet, but the multitude of active substances (Figure 2) and the wide therapeutic potential of this plant are a great challenge for researchers around the world.

## 3. Methodology

The PubMed and Google Scholar databases were searched using combinations of the keywords: Ashwagandha, *Withania somnifera*, withanolides, endocrine system, HPA axis, HPG axis, thyroid, TSH, reproductive system, testosterone, cortisol, hormones, and infertility. The search mainly included research published in the years 2010–2023.

## 4. Regulating Hypothalamus, Pituitary Gland and Their Axis by Ashwagandha

Is it difficult to discuss the pituitary gland and hypothalamus as separate glands of the hormonal system. Together, they create a complex system of neuroendocrine pathways and feedback loops which maintain homeostasis.

Hypothalamus as an overriding hormonal gland, manages the activity of the pituitary gland due to its neurotransmitters and hormones it releases. In turn, the pituitary is made up of two lobes (anterior—secrets hormones produced in this gland; and posterior—releases hormones produced in hypothalamus). In this way, it controls such organs as kidneys by vasopressin (ACTH), breasts and uterus by oxytocin, also ovaries and testes trough the secretion of gonadotropins—lutropin (LH) and folliculotropin (FSH). It regulates the level of corticoids due to adrenocorticotropin (ACTH) secretion and its action on adrenal glands, breast by prolactin (PRL), liver, bones and muscles by growth hormone (GH), skin by melanotropin (MSH), lipid tissue by lipotropin (LPH) and finally regulates thyroid gland activity by thyrotropin (TSH).

Multiplicity and multidirectional actions of secreted hormones make any imbalance visible in consequences like infertility, menstrual disorders, growth disorders, weakness, somnolence, dysregulated blood pressure and others dependent on which pathways and feedback loops are dysregulated. Due to the above information, we would like to summarize how Ashwagandha and its active substances regulate the hypothalamus and pituitary gland activity, and the influence on the axis which they create with other endocrine glands.

The studies have shown that *W. somnifera* can exhibit its effect at the hypothalamic level. Kataria et al. isolated the hypothalamic clonal cell line, GnV-3 cells, which are GnRH (gonadotropin-releasing hormone) neurons isolated from rat brain. They proved that aqueous extract from Ashwagandha’s leaves stimulates GnRH cells activity which results in increased GnRH release. Based on in vitro studies on mice hypothalamus slices, it was established that *W. somnifera* can exhibit GABA-mimetic action due to its interactions with GABA type A receptors [40]. Thus, it leads to increased secretion of GnRH in hypothalamus and that is the way Ashwagandha can modulate HPG axis at the hypothalamic level [12]. On the other hand, the other studies investigated how the GABAA receptor antagonists block the ability of methanolic root extract of Ashwagandha to induce depolarization in mice GnRH neurons in juvenile mice. Scientists depolarized GnRH neurons by bath application of Ashwagandhas methanolic extract. Using the patch clamp method, they proved that the *W. somnifera*-induced inward currents were suppressed by bicuculine methiodide—a GABAA receptor antagonist. In the above mentioned studies, the researchers came to the same conclusion that releasing GnRH can be modulated by GABA-mimetic action of *W. somnifera* [41].

To supplement the above, it should also be noticed that GABAA receptors are the main target of anti-anxiety drugs, so it can therefore be assumed that Ashwagandha reduces stress levels through that mechanism [21]. Also high cortisol relation with the HPA (hypothalamic–pituitary–adrenal axis) response to stress is well known. Many studies confirmed that illnesses like depression or anxiety disorders are associated with dysregulation of this axis, which results in increases cortisol and DHEA (dehydroepiandrosterone) levels. Inversely, there are also studies, which mention that high DHEA concentrations indicate high exposure to stress or overactivity of the HPA axis, which forms a coherent whole [2,21]

Lopresti et al. performed a 60-day randomized, double-blind, placebo-controlled trial, during the 15-day intake of 240 mg of *W. somnifera* extract in mildly anxious, healthy sixty adults. The extract was standardized by high-performance liquid chromatography to contain 35% withanolide glycosides—approximately 84 mg withanolide. Participants were instructed to take 1 capsule (with 84 mg withanolide glycosides), once daily after dinner with 250 mL of water. Such therapy resulted in significant emotional improvement over time, measured by reduction in HAM-A score (Hamilton Anxiety Rating Scale) and reduction in morning cortisol and DHEA-S (dehydroepiandrosterone sulfate) levels. Based on the HAM-A score, they deduced that anxiety level reduced by 41% in the test group and only by 24% in the placebo group. These conclusions were further confirmed by positive improvements in DASS-21 (Depression Anxiety Stress Scale—21)—30% in the study group vs. 10% in the placebo group. As the explanation of these results, authors suggested that Ashwagandha may have an attenuating effect on the HPA axis activity, what was confirmed by the decreased level of fasting morning cortisol level (0.5% decrease in placebo group and 23% decrease in the study group) and DHEA-S (2.5% increase and 8.2% decrease, respectively) in male participants. Statistically significant changes in levels of these hormones occurred in both sexes [16]. Another research group came to the same conclusions by conducting an 8-week double-blind, placebo-controlled study in which 60 participants were divided into three groups—a control group, one group receiving 250 mg of Ashwagandha root extract per day and the third group receiving 600 mg/day of Ashwagandha root extract. As a stress gauge, serum cortisol level was used, measured at a baseline, at 4 weeks and at 8 weeks of treatment; HAM-A was used to assess the intensity of anxiety, the Perceived Stress Scale (PSS) score and a sleep quality was measured by a 7-point scale questionnaire. In both study groups, serum cortisol levels were significantly lower than in placebo group at the eighth week. Also, the improvement in anxiety reduction and sleep were noticed. The observed effects were dose dependent [42].

An interesting study was conducted by Mahdi et al. on a group of men with unexplained infertility, some of whom smoked cigarettes in the past (they were under environmental stress) and some were under constant mental stress. All of them were given 5 g of Ashwagandha per day orally with a cup of milk for three months. As a result, the levels of LH, testosterone and antioxidants increased and a decrease in seminal LPO (lipid peroxidation), stress and serum cortisol level was observed, which resulted in increased pregnancy rates in the partners of the subjects. According to the authors, stress leads to low testosterone levels due to a reduction in the LH pulse frequency, so conclusions were drawn that Ashwagandha reduces stress, which in turn improves fertility by modulating the HPG axis [43].

Analogous inferences were proposed by the authors of an equine model—horses which received Ashwagandha root extracts (containing >5% withanolides) had lower levels of cortisol and epinephrine after stress-inducing exercises than the placebo group. Scientists concluded that stress activates the HPA axis and may induce a secretion of cortisol and epinephrine, also diminishing the serotonin levels. As a result, Ashwagandha reduces stress by lowering the stress hormone levels [44]. Table 3 shows the results of the studies discussed above.

Some reports mention that steroids which Ashwagandha roots contain act like exogenous adrenocortical steroids and decrease ACTH secretion, which results in lowering of endogenous steroids synthesis. Even bolder conclusions have been drawn that this influence of *W. somnifera* on endogenous steroids makes Ashwagandha to be considered a growth promoter, particularly during development [45].

Steroid levels have an influence on mood, sexuality, the immune system, blood pressure, growth and many other important components of human health. It seems that Ashwagandha and its active compounds, due to the modulating impact on hormonal axis, can be promising in maintaining overall human wellness. In turn, considering the fact that we are all constantly exposed to mental or environmental stress, which results in fluctuations in the functioning of the hormonal axes—crucial for homeostasis—Ashwagandha seems to be an extremely interesting research subject due to the above-mentioned properties.

## 5. Ashwagandha’s Impact on the Reproductive System

Ashwagandha has been used as an aphrodisiac in Ayurveda for thousands of years. Nowadays, researchers are intensively exploring the potential of *W. somnifera* as a possible remedy for reproductive system disorders. One of the major reproductive health problems is infertility. The WHO defines it as the inability to achieve pregnancy following 12 months of regular sexual intercourse without contraception. It is estimated that approximately 8 to 12 percent of couples and 186 million individuals around the world are affected by this condition [46]. Infertility is a serious social, emotional and demographic problem [2], so there is an urgent need to find a good solution. Male and female factors contribute almost equally to the causes of infertility [47,48].

In the female reproductive system, the causes of infertility can be grouped into a few broad categories such as tubal, uterine or ovarian disorders and endocrine abnormalities. Similarly, the main categories in men are problems with ejaculation, testicular failure to produce sperm, abnormal volume and/or quality of semen and hormonal disorders. This chapter outlines some of the possible ways in which *W. somnifera* may affect reproductive health.

As mentioned earlier, acting on the HPG axis and thus affecting sex hormone balance appears to be an essential modulation. A plausible mechanism may be that Ashwagandha acts on GABA receptors in the hypothalamus, facilitating the expression of GnRH [49], which in turn stimulates the pituitary gland to secrete LH and FSH [50,51].

Another study investigated the efficacy of *W. somnifera* use in an animal model of infertility—the photorefractory Japanese quail with regressed testes and decreased expression of estrogen receptor alpha [51]. The decreased expression of estrogen receptor alpha in the testes of photorefractory quail was increased after oral administration of *W. somnifera* root extract compared to the control. It was found that *W. somnifera* root extract alleviates the reproductive inactivity or photorefractory state of quail by activating the HPG axis and estradiol secretion.

In another animal model study, the efficacy of single or combined administration of matcha and Ashwagandha tea against H_2_O_2_-induced utero-ovarian oxidative injury and cell death in female rats was investigated. Administration of hydrogen peroxide to female rats caused a significant reduction in serum FSH, LH, progesterone and estrogen levels compared to a healthy control group. Supplementation of injured rats with matcha tea alone, Ashwagandha tea alone or in combination significantly improved these parameters and significantly restored estrous cycle length compared to the injured control [52].

Testosterone is produced mainly by Leydig cells in the testes under the influence of luteinizing hormone. To date, several studies have shown that supplementation with Ashwagandha leads to a significant increase in testosterone levels [46,49,50,53,54].

The 8-week randomized, double-blind, placebo-controlled trial investigated the effects of Ashwagandha root extract in adult men with reduced sexual desire. In the study group, participants took 300 mg of Ashwagandha root extract twice daily. Serum testosterone and serum prolactin levels were assessed at baseline and week 8. Results were within the optimal range for all participants at both visits. There was a statistically significant increase in serum testosterone in the Ashwagandha group compared to baseline. Ashwagandha root extract supplementation was also associated with a statistically significant increase in serum testosterone levels compared to placebo, whereas non-significant changes in serum prolactin levels were observed in both groups. The authors also noted that withanolides are steroidal lactone triterpenoids that are chemically similar to testosterone and are therefore thought to provide the benefits of male steroid hormones [49].

The systematic review by Smith et al. looked at the effects of different herbs on testosterone levels in men. In addition to fenugreek seed extracts, Ashwagandha root and leaf extracts were found to be the most effective. The four trials included in this systematic review considered the effect of Ashwagandha on testosterone levels. Three of them showed positive effects of Ashwagandha supplementation on testosterone concentration in men, while one trial showed no effect of supplementation. The authors also point out that classic testosterone replacement therapy (TRT) has many adverse effects and contraindications, so there is a need to look for alternatives, and one of these could be herbal medicines such as Ashwagandha [54].

A different systematic review, by Gómez Afonso et al., evaluated variations in testosterone, DHEA and cortisol levels in healthy adult subjects receiving *W. somnifera* extracts in different dosing patterns. Overall, participants supplemented with *W. somnifera* showed significant improvements in testosterone in most of the studies analyzed. All the trials that looked at cortisol levels found significant reductions in this hormone. For DHEA, the two trials included in the analysis showed conflicting results [53].

Speaking of cortisol, we need to take a closer look at this issue. Ashwagandha’s adaptogenic and stress-reducing properties have been known for thousands of years. By acting on the HPA axis, it causes cortisol levels to fall. This fact is crucial for the physiological function of the reproductive system, as high levels of cortisol interfere with the HPG axis [51,55]

Some authors suggest that cortisol, the body’s main stress hormone, is inversely correlated with testosterone levels, and that reducing its production may increase testosterone levels. In several human studies, Ashwagandha supplementation has been associated with reduced cortisol concentrations, which may contribute to the testosterone-enhancing effects [54].

Antioxidative properties of Ashwagandha have been proved in numerous studies before. Ashwagandha’s antioxidant potential is due to phytochemical constituents such as flavonoids and phenolic compounds [56]. It has been suggested that reactive oxygen species may contribute to infertility in a variety of ways, for example by interfering with spermatogenesis or sperm-ovum mating [50]. Thus, neutralization of ROS may be an important mechanism by which Ashwagandha improves fertility.

Another study investigated the antioxidant capacity and in vitro response of phytochemical constituents of *W. somnifera* on standard parameters of semen from healthy volunteers [56]. Incubation of semen with *W. somnifera* extracts improved sperm motility and preserved sperm viability for a longer period than the control. The study concludes that *W. somnifera* has favorable in vitro properties that may aid in the preservation of spermatozoa during intrauterine insemination and in vitro fertilization.

In some articles, the authors also assessed patients’ subjective sexual performance using different scales. For example, one study aimed to evaluate the efficacy and safety of standardized Ashwagandha root extract (300 mg twice daily) in improving sexual function in healthy women. Participants were otherwise healthy women with hypoactive sexual desire disorder (HSDD; with a Female Sexual Function Index (FSFI) score <26 or Female Sexual Distress Scale (FSDS) score >11). There was a statistically significant improvement in FSFI scores with Ashwagandha compared to placebo at 8 weeks, and this improvement was observed in all subscales (desire, arousal, lubrication, orgasm, sexual satisfaction and pain) of the FSFI scale. There was a greater improvement in FSDS scores with Ashwagandha compared to placebo. More women with Ashwagandha reported improvements in satisfying sexual encounters (SSEs) at week 4 and week 8 compared to placebo [14].

In another study mentioned in part above, 50 healthy male subjects with low sexual desire (as measured by a score of 15 or less on the sexual desire domain of the DISF-M questionnaire) were randomized to take 300 mg of Ashwagandha root extract or placebo capsules twice daily. Outcomes were measured using the Derogatis Interview for Sexual Functioning-Male (DISF-M) questionnaire and the Short Form Survey-36 Quality of Life questionnaire before and after the intervention. Ashwagandha root extract increased participants’ ability to perform better in all the five DISF-M domains, including sexual cognition, sexual arousal, sexual behavior, orgasm, and sexual desire. A statistically significant increase in the total DISF-M score was observed over time in the Ashwagandha and placebo groups. When comparing the Ashwagandha group with the placebo group, a statistically significant improvement in the total DISF-M score in the Ashwagandha group was noted. The Ashwagandha group saw a 40% increase in total DISF-M score compared to a 25% increase in the placebo group. This study also supported the improvement and maintenance of quality of life in participants taking the Ashwagandha supplement [49]. Table 4 shows the results of the studies discussed above.

**Figure 3:**
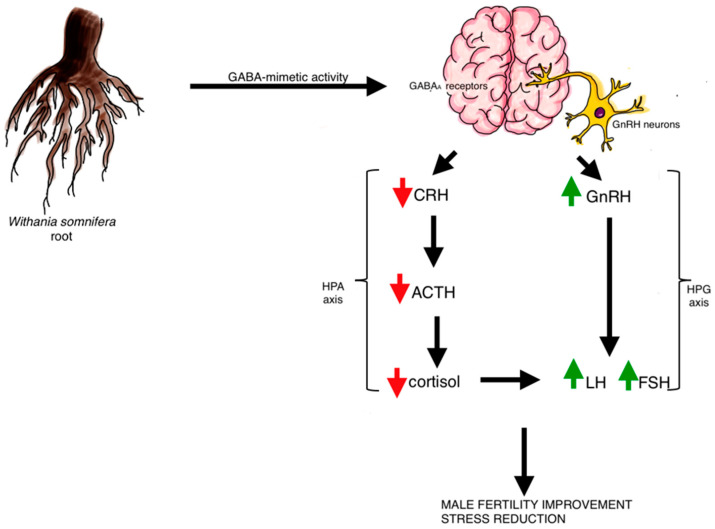
Due to its active substances *W. somnifera* acts through GABAA receptors. It causes increased GnRH release, which results in expanded gonadotropins release. It also reduce cortisol level. These interactions have an influence on stress reduction and improving male fertility.

This interesting topic of the influence of Ashwagandha preparations on the reproductive system still needs intensive research. The lack of clinical trials on large groups of patients makes it difficult to draw clear conclusions in this field. Most of the studies conducted so far have involved relatively healthy participants and small groups. Ashwagandha seems to be a promising starting point for the future development of drugs for reproductive system disorders, and furthermore, research to date has not reported many serious adverse events from the administration of Ashwagandha. In our opinion, future research should pay more attention to the mechanisms of neurohormonal regulation of reproduction. Studies should also evaluate the potential interactions of Ashwagandha with drugs already used in the treatment of reproductive disorders. Figure 3 shows the suggested mechanism of action of Ashwagandha discussed in the above sections.

GABAA receptor—gamma aminobutyric acid type A receptor; CRH—corticotropin-releasing hormone; ACTH—adrenocorticotropic hormone; GnRH—gonadotropin-releasing hormone; LH—luteinizing hormone; FSH—follicle—stimulating hormone; HPA axis—hypothalamic–pituitary–adrenal axis; HPG—hypothalamic–pituitary–gonadal axis.

## 6. Ashwagandha and Thyroid Gland Dysfunctions

The thyroid gland and the hormones it synthesizes, such as triiodothyronine (T3) and thyroxine (T4), play a key role in metabolism, reproduction and the proper functioning of the human body. Thus, pathological conditions affecting the thyroid affect many other systems, which may have an incalculable number of effects on our health.

Ashok Kumar Sharma et al. in their double-blind, randomized, placebo-controlled study studied patients with subclinical hypothyroidism (SCH). SCH is defined as thyroid dysfunction as indicated by a high TSH level with a normal T4 level. In the case of SCH, clinical symptoms of hypothyroidism such as poor cold tolerance, drowsiness, fatigue or hair loss may or may not be present. Over 8 weeks in a group of 50 patients, half was given Ashwagandha root extract (300 mg twice a day) and the other half was given a placebo (starch). During the study, material was collected for monitoring of TSH, serum T3 and T4 levels. By evaluating the initial results and comparing them with those during the study, the authors observed a decrease in TSH, and increase in T3 and T4 levels in the study group. On the other hand, in the control group, the levels of T3 and T4 were either lower than the baseline values or remained at the same level. The largest increase in the study group for triiodothyronine level in serum was observed in the 4th (+18.6%) and 8th (+41.5%) week of Ashwagandha supplementation. In the case of T4, the increase was 9.3% in the 4th week and 19.6% in the 8th week of the study. In addition, the TSH level decreased significantly after 4 weeks of the study (−12.5%) and (−17.4%) after 8 weeks. The above study may suggest that Ashwagandha supplementation in patients with subclinical hypothyroidism may be beneficial in the treatment of this condition. More studies are needed, both in patients with SCH and other dysfunctions affecting the thyroid gland, to fully confirm the benefits of Ashwagandha supplementation and the safety of its use [13].

Khaled G. Abdel-Wahhab et al. studied the effect of Ashwagandha methanolic extract on a rat model with induced hypothyroidism. During the study, specific groups of rats received either Ashwagandha methanolic extract (500 mg/kg/day) or levothyroxine (20 μg/kg), a clinically approved drug for the treatment of hypothyroidism. What is worth mentioning, both groups showed not only similar results in thyroid tests (TSH, total T3, free-T3, total T4 and free-T4) but also in the subsequent histopathological examination. And most important, both groups expressed above showed significantly better thyroid results than the non-treated controls [57].

The above results are not only promising, but also repeatable, which was confirmed in a similar study by Noha Abdellatif Ibrahim et al. In this project, researchers also induced hypothyroidism in a group of rats. The group of rats which they administered only propylthiouracil (PTU) had significantly lower T3 and T4 values and higher TSH values than the group of PTU + Ashwagandha rats, which confirms the “protective” effect of *W. somnifera* substances on the thyroid gland. What is even more interesting, the authors point out the lack of toxicity and safety of using Ashwagandha in their study. The administration of 50 mg/kg/day did not cause any toxic effects in any of the rats, which gives 100% survival for the time of administration [58]. Table 5 shows the results of the studies discussed above.

Summing up, thanks to the above studies, it can be concluded that the effect of Ashwagandha is promising not only due to the benefits of Ashwagandha supplementation on the thyroid gland, but also the relative safety of use [40]. However, it should be noted that cases of thyrotoxicosis have been described that may be associated with excessive use of *W. somnifera* derivatives [59,60], like a 62 y.o. patient who developed thyrotoxicosis by supplementing 1950 mg of Ashwagandha root extract a day for approximately two months [59]. What is worth mentioning is that there is still more work to be performed. Researchers should pay attention to the mechanism of action of Ashwagandha. It is assumed that the possible effect of the action on the thyroid hormones results from the direct stimulation of the thyroid gland to the secretion of T3 and T4, and as a result, a decrease in TSH, which is a consequence of the hypothalamic–pituitary–thyroid (HPT) axis. Also to broaden the topic, more research on humans should be conducted, taking into account the causes of hypothyroidism as well as other diseases related to the organ such as the thyroid gland.

## 7. Limitations

Despite numerous in vitro and in vivo preclinical studies, there is a need for clinical trials to confirm the therapeutic properties of *W. somnifera* extracts. The results so far confirm that Ashwagandha is a promising drug candidate for many conditions. The study showed that participants who were administered Ashwagandha root extract for 8 weeks showed no adverse effects. Ashwagandha root extract was proven to be safe and well tolerated [61]. The widespread use of Ashwagandha carries the risk of not controlling other therapies the patient is receiving. It is worth remembering that the plant itself also has many contraindications. For example, in patients with hyperthyroidism, it can exacerbate the effects of the disease—irritability, restlessness, nervousness, anxiety, hand tremors, palpitations, psychomotor agitation, etc.—by increasing the levels of T3 and T4 [2]. To objectify our opinion about Ashwagandha, we delved even deeper into research on its safety. One of the studies presents 8 cases (6 males and 2 females) of Ashwagandha-induced liver injury. This study seems to be reliable, because it included patients using only Ashwagandha and excluded patients who consumed Ashwagandha as one of multiherbal preparation ingredient, as well as patients using alcohol or those with other causes of acute liver injury. Three patients had pre-existing liver injury and two of them were diagnosed to have underlying chronic liver disease simultaneously with the diagnosis of herb-induced liver injury (HILI). The predominant described type of Ashwagandha-induced liver injury was cholestatic (in four patients), other types were hepatocellular (in three) and mixed (in one). Three of the patients with Ashwagandha-induced liver injury and underlying liver disease died during the follow-up [62]. Our bold conclusion from this cohort study is that *W. somnifera* should not be used in people with coexisting liver disorders, at least no arbitrarily without medical supervision.

An important issue in the context of the development of preparations containing *W. somnifera* extract is the standardization of the raw material and the development of an appropriate formula with high bioavailability. Studies have shown that withaferin A is characterized by low bioavailability, which may translate into a low therapeutic effect [63]. It seems that a promising formulation for this plant are sustained-release (SR) capsules, which not only increase the bioavailability of active substances, but also improve compliance. In a comparative pharmacokinetic study, Gopukumar et al. showed that the relative bioavailability of Ashwagandha SR was 12-, 44- and 11-fold higher for all withanolides, withanolide A and 12-deoxyvitastramonolide, compared to the standard release reference formulation, respectively [64]. It seems, therefore, that in the case of Ashwagandha, the important issue is to create the right formula to fully realize the therapeutic potential of its active ingredients.

As mentioned earlier, Ashwagandha has been widely used in traditional medicine for thousands of years, but it is worth considering that modern translational research reveals a reasonable interpretation in the context of traditional uses. In addition, new research also explains its pharmacological mechanisms and chemical biology [4]. Interestingly, Ayurveda used different pharmaceutical forms, such as juice, paste, decoction, cold infusion and hot infusion [65]. Currently, Ashwagandha is mainly used in the form of capsules or tablets, containing powdered extract of its root [4]. It seems obvious that, in the case of traditional and current medicine, there was a difference in the dose used, which could determine the effect of this plant and its safety of use. The studies we discussed in this review, show that the currently most common dosage regimen for Ashwagandha in humans is 300 mg twice daily. This dose seems to be effective and safe.

## 8. Conclusions

In conclusion, Ashwagandha is a remarkable medicinal plant renowned for its diverse therapeutic properties. These properties are attributed to the wealth of active substances found within the plant, making it a subject of extensive research and interest.

Of special interest is Ashwagandha’s influence on the endocrine system. It has been shown to modulate pituitary functions, improve thyroid gland homeostasis, regulate adrenal activity, and exert a multifaceted influence on the reproductive system. Some studies have assessed subjective sexual performance, reporting improvements in sexual desire, arousal, satisfaction, and overall quality of life in both women and men taking Ashwagandha supplements. Ashwagandha’s stress-reducing properties and its ability to lower cortisol levels are crucial in the context of reproductive health. High cortisol levels can interfere with the HPG axis, making the reduction in cortisol production a valuable aspect of enhancing reproductive function. This diverse range of effects can be attributed to the multitude of bioactive compounds present in various parts of the Ashwagandha plant.

Due to the fact that research conducted on humans cannot deprive people of their right to effective treatment, we are not able to precisely assess the impact of substances derived from *W. somnifera* on the endocrine system, but only draw conclusions about their potentially beneficial or unfavorable effect. Additional factors such as comorbidities, medications taken or potential individual differences are often not taken into account due to the lack of information in research. The safety and toxicity of Ashwagandha also remain important issues, which may affect its potential use in specific patient groups.

In light of these findings, it is clear that Ashwagandha holds significant promise as a natural remedy for various health concerns, especially those related to the endocrine system. Future research may provide new insights into its mechanisms of action and expand its applications in both traditional and modern medicine.

## Figures and Tables

**Table 1:** Classification of the active substances in *W. somnifera* (data from Gaurav H., 2023 [22]).

Compounds	Part of *W. Somnifera*	Active Substances
Alkaloids	Leaves, roots, stems	Ashwagandhine, anahygrine, anaferine, pseudotropine, tropine, isopelletierine, [3]-tigloyloxtropine, tropeltigloate, dlisopelletierine, hygrine, mesoanaferine, choline, somniferine, withanine, withananine, hentriacontane, visamine, withasomnine, somniferinine, somninine, nicotine, cuscohygrine
Flavonoids	Roots, stems	Quercetin, 7-hydroxyflavone kaempferol
Glycosides	Roots, stems	Withanosides I–VII, withanamides
Phenolic	Roots, stems	Coumaric acid, caffeic acid, chlorogenic acid, gallic acid, ferulic acid, catechin
Saponins	Roots, berries	Sitoindoside VII, sitoindoside VIII
Steroids	Roots	β-sitosterol, cholesterol, diosgenin, ergostane, sitoindosides IX, X, stigmastadien, stigmasterol
Steroidal lactones	Leaves, roots	Withaferin–A, withanone, withasomidienone, withanolides A-Y, withasomniferine, withasomniferols A-C
Tannins	Roots, leaves, fruits, flowers	Not available

**Table 2:** Potential therapeutic effects of some active substances found in Ashwagandha.

Active Substance	Potential Effects	References
Anaferine	anti-tuberculous,	[27]
	neuroprotective	[28,33]
Anahygrine	anti-tuberculous,	[27]
	neuroprotective	[29,30]
Withaferin A	anti-inflammatory,	[24]
	anti-cancer,	[19,24,31,32]
	anti-diabetic,	[25]
	cardioprotective,	[25]
	neuroprotective,	[25,28,33]
	antibacterial,	[34]
	anti-SARS-CoV-2,	[35,36]
	in dermatological diseases	[37]
Withanolide D	neuroprotective,	[28,33]
	anti-cancer	[20]
Withanone	antibacterial,	[34]
	anti-SARS-CoV-2,	[35,36]
	anti-cancer	[19]

**Table 3:** Ashwagandha’s effects on hypothalamus, pituitary gland and their axis—a summary of the studies.

Authors	Characteristic of the Group	Ashwagandha Formulation Characteristics	Duration of the Observation	Results	References
Lopresti et al.	stressed healthy males and females 18–64 years old, with a HAM-A between 6 and 17	240 mg of Ashwagandha extract per day for 15–days, standarized contain 35% withanolide glycosides—approximately 84 mg withanolide; oral administraion	60 days after commencement of 15-day capsule intake	↓cortisol ↓DHEA-S ↑testosterone level in males - emotional improvement ↓anxiety measured in HAM-A score and DASS-21	[16]
Salve J et al.	60 participants (males and females), divided into three groups	one study group receiving 250 mg of Ashwagandha root extract per day; another study group receiving 600 mg of Ashwaganda extract per day; oral administraion	8 weeks	↓anxiety ↓cortisol - sleep improvement	[42]
Mahdi et al.	121 men, 25–38 years. 60 men with unexplained infertility under environmental or constant mental stress in the study group, divided into three subgroups	5 mg of Ashwagandha root powder for three months; oral administration	The patients were followed for partner’s pregnancy outcome for a period of 3 months after the treatment	↑LH ↑testosterone ↑antioxidants ↓stress ↓cortisol	[43]
Priyanka G et al.	24 healthy Kathiawari horses of either sex, 5–10 years old, divided into four groups: one control group and three experimental groups given varying dose of Ashwagandha	high-concentration full-spectrum Ashwagandha root powder, containing ≥5% of withanolides; experimental groups were given a varying dose of Ashwagandha (2.5 mg/animal, 5 mg/animal, and 10 mg/animal) with jiggery, respectively; oral administration	21 days of intake, after 14 days of intake horses were subjected to different kind of stress	↓cortisol ↓epinephrine ↑serotonine	[44]

**Table 4:** Ashwagandha’s effects on the reproductive system—a summary of the studies.

Authors	Characteristic of the Group	Ashwagandha Formulation Characteristics	Duration of the Observation	Results	References
Baghel et al.	18 sexually mature six weeks old male Japanese quail as a animal model of infertility, provoked by using photoperiodic chambers	100 mg/day/kg of *W. Somnifera* root extract; oral administraion	few months of inducing infertility using photoperiodic chambers and 45 days of Ashwagandha administration	↑expression of estrogen receptor alpha ↑estrogen ↓corticosterone	[51]
Megahd et al.	50 adult female rats; model of H_2_O_2_-induced utero-ovarian oxidative injury and cell death, what caused reduction in serum level of FSH, LH, progesterone, and estrogen compared to a healthy control group	200 mg/kg Ashwagandha tea extract; oral administraion	1 month	↑FSH ↑LH ↑progesterone ↑estrogen -significantly restored estrous cycle length	[52]
Ajgaonkar et al.	prospective, randomized, placebo-controlled study; 80 women, 18–50 years old, without any hormonal disturbances and having hypoactive sexual desire disorder (HSDD) with a Female Sexual Function Index (FSFI) score < 26, or Female Sexual Distress Scale (FSDS) score > 11	300 mg of standardized Ashwagandha root extract twice daily; oral administraion	8 weeks	-improvement in sexual functions	[14]
Chauhan et al.	randomized, controlled trial; 50 healthy male subjects with low sexual desire	300 mg of Ashwagandha root extract twice daily; oral administraion	8 weeks	↑testosterone -improvement of sexual functions measured by DISF-M	[49]

**Table 5:** Ashwagandha’s effects on thyroid gland—a summary of the studies.

Authors	Characteristic of the Group	Ashwagandha Formulation Characteristics	Duration of the Observation	Results	References
Sharma et al.	males and females, 18–50 years old, with subclinical hypothyroidism	300 mg of Ashwagandha root extract twice a day; oral administration	8 weeks of treatment	↑ T3 ↑ T4 ↓ TSH	[13]
Abdel-Wahhab et al.	male albino rats with induced hypothyroidism	500 mg/kg/day of Ashwagandha methanolic extract; oral administraion	30 days	↑ T3 ↑ fT3 ↑ T4 ↑ fT4 ↓ TSH	[57]
Ibrahim et al.	male Wistar albino rats with induced hypothyroidism	50 mg/kg/day of Ashwagandha extract; oral administraion	30 days	↑ T3 ↑ T4 ↓ TSH	[58]
